# Chemical composition of Egyptian propolis and studying its antimicrobial activity and synergistic action with honey against some multidrug-resistant uropathogens

**DOI:** 10.1038/s41598-025-00773-1

**Published:** 2025-05-20

**Authors:** Asmaa K. Helmy, Nagwa M. Sidkey, Marwa M. Abdel-Aziz, Atef A. El-Hela

**Affiliations:** 1 Botany and Microbiology Department, Faculty of Science for Girls, Al-Azhar University, Cairo, Egypt; 2 The Regional Centre for Mycology and Biotechnology, Al-Azhar University, Cairo, Egypt; 3 Pharmacognosy and Medicinal Plants Department, Faculty of Pharmacy, Al-Azhar University, Cairo, Egypt

**Keywords:** Uropathogens, Propolis, Antimicrobial activity, EEP, Ethanolic extract of propolis, Active fraction, GC-MS, HPLC, Antimicrobials, Microbiology techniques, Chromatography

## Abstract

Urinary tract infection (UTI) is one of the most common infections worldwide, increasing the incidence of antibiotic resistance and creating demand for alternative antimicrobial agents. Propolis, a natural antimicrobial agent, has been used in ancient folk medicine. This study evaluates the effectiveness of ethanolic extract of propolis (EEP) alone and in combination with honey against multidrug-resistant (MDR) uropathogens and also investigates the chemical composition of Egyptian propolis, which may be a potential therapeutic approach against MDR uropathogens. EEP was prepared, followed by column chromatographic fractionation using four different solvent systems. The ethyl acetate fraction was further fractionated through vacuum liquid chromatography (VLC). The antimicrobial activity of the EEP, propolis fractions, honey, and EEP-Honey mixture was studied, and the fraction with the best antimicrobial activity was analyzed by GC-MS and HPLC. The results indicated that EEP showed antimicrobial activity against the five MDR uropathogens with varying potential, while honey showed no activity against these pathogens. In comparison, the EEP-Honey mixture exhibited good antimicrobial synergy, with the MIC value decreasing by approximately 4–8 folds. In propolis fractionation, ethyl acetate was the best solvent for extracting antimicrobial substances from EEP, and fraction 5 (F5) was the most active fraction, with inhibition zone diameters of 30.33, 29.00, 21.58, 25.33, and 27.67 mm against MDR *P. aeruginosa*, *E. coli*, *K. pneumoniae*, *S. saprophyticus*, and *C. albicans*, respectively. GC-MS analysis of the F5 fraction revealed the presence of phenolics, flavonoids, terpenoids, acids, and alkaloids. In addition, HPLC polyphenol analysis identified 14 phenolic acids and flavonoid compounds with concentrations ranging from 117.36 to 5657.66 µg/g. Overall, the current findings highlighted the promising antimicrobial synergy of the EEP-Honey mixture against MDR urinary pathogens. The phytochemical analysis of propolis also identified potential bioactive compounds responsible for its biological and pharmaceutical properties.

## Introduction

Urinary tract infections (UTIs) are among the most well-known community and hospital-associated microbial infections, affecting more than 150 million people annually worldwide^1,2^. They are among the most frequent complications in critical care patients^2,3^. Generally, antibiotics are prescribed as empiric therapy even as the result of urine culture, and this approach has probably contributed to the increase in antibiotic resistance worldwide^4^. Thus, the improper use of antibiotics may delay effective treatment and participate in the emergence of multidrug-resistant (MDR) microbes^5,6^. The World Health Organization (WHO) and other researchers have emphasized the urgent need for novel antimicrobial approaches to combat infectious diseases^7–9^.

Propolis, or bee glue, is a naturally occurring sticky material that belongs to the family of bee products^9,10^. The word “propolis” originates from two ancient Greek words: *pro*- means “in front of” or “at the entrance,” and *polis* means “city” or “community,” collectively referring to a substance used to defend the hive^10,11^. Propolis is collected by worker bees from the resinous secretions of a variety of plant species, such as pine, poplar, alder, conifer, beech, and birch, and then mixed with enzymatic and salivary bee secretions to form bee glue^12,13^.

Propolis has been used in folk medicine since ~ 300 BC and is reported to have several biological activities, including antibacterial, fungicidal^9,14^, antiviral, immunomodulator^15,16^, anti-inflammatory^17^, antioxidant^17,18^, and antitumor^19^. Furthermore, a previous study has demonstrated the good effect of propolis in increasing the growth rate of some types of probiotic bacteria^20^.

Indeed, the raw propolis consists of 45 to 55% plant resin, 25 to 35% beeswax, 5 to 10% aromatic and essential oil, 5% pollen, and 5% other natural constituents^21^. Propolis also contains various sorts of secondary plant metabolites, such as phenolic acids, tannins, terpenoids, alkaloids, and flavonoids, which are responsible for several bioactivities and differ in concentrations depending on the season, plant sources, and geographical origins^9,22,23^. It has many targeted sites with strong activity, attributed to its phenolic constituents, so it can potentially increase the effectiveness of other antimicrobial agents and act synergistically^24^. The current study aimed to investigate the antimicrobial activity of the crude ethanolic extract of propolis (EEP), its synergistic effects when combined with honey, and the chemical composition of propolis responsible for its antimicrobial properties.

## Materials and methods

### Preparation of propolis extract, honey, and their mixture

A raw Egyptian propolis sample was obtained from the Abd El-Raheam apiary located in Marsa Matrouh, Egypt, and stored in a sterile glass container in the refrigerator, away from sunlight, until extraction. It was extracted with 70% ethanol (1:10, w/v), according to Helmy et al.^9^. The sample was incubated under agitation for seven days at 37 °C, protected from light, followed by centrifugation for 10 min. The supernatant was dried at 40 °C until ethanol evaporation occurred to obtain the pure propolis extract in powder form. This powder was weighed and dissolved in 1% DMSO (Sigma-Aldrich) to obtain EEP at a concentration of 100 mg/ml.

An Egyptian Citrus honey sample was obtained from the Egyptian Ministry of Agriculture, Giza, Egypt, and stored in a sterile glass container at room temperature. It was prepared in a 50% (v/v) concentration by adding an equal volume of honey to an equal volume of sterile distilled water in a sterile test tube. The honey solution was mixed by stirring with a vortex.

A mixture of EEP and honey (EEP-Honey) was prepared (1:1 v/v) to get a mixture of the final concentration (100 mg EEP/ 50% honey) to study the antimicrobial synergism between propolis and honey.

### Tested uropathogens

Five MDR uropathogens were used in the current study, including one yeast isolate: fluconazole-resistant *Candida albicans*, one strain of Gram-positive bacteria: oxacillin and mecillinam-resistant Coagulase Negative (CON) *Staphylococcus saprophyticus*, and 3 Gram-negative MDR bacteria included extended-spectrum beta-lactamases (ESBL) *Klebsiella pneumoniae*, as well as MDR *E. coli* and *Pseudomonas aeruginosa*, which resisted to ceftazidime, cefepime, aztreonam, ciprofloxacin, piperacillin, imipenem, and gentamicin.

All urinary pathogens were provided by the Clinical Microbiology Laboratory of Cleopatra Hospital, Cairo, Egypt, from urine and catheter specimens whose patients were suffering from UTIs. These pathogens were identified according to the VITEK 2 system. VITEK 2 (bioMérieux) is an automated, highly accurate microbial identification system based on phenotypic identification methods. This system accommodates colorimetric reagent cards that are automatically incubated and interpreted. Four reagent cards are used for the identification of different microorganisms: (GN) for Gram–negative bacteria; (GP) for Gram–positive cocci and non-spore-forming bacilli; (BCL) for Gram–positive spore-forming bacilli; and (YST) for yeasts and yeast-like microorganisms. Each reagent card contains 64 wells, each containing an individual test substrate for measuring different metabolic activities^25^.

### Antimicrobial activity of EEP, Propolis fractions, and honey

#### Agar well diffusion method

Antimicrobial activities of EEP, propolis fractions, honey, and DMSO were determined by the agar well diffusion method on Mueller-Hinton agar (Oxoid, UK) for bacteria and on 2% glucose-supplemented Mueller-Hinton agar for *Candida* spp^26^. This test was carried out in the Biotechnology Lab, Faculty of Science, Al-Azhar University for Girls, Cairo, Egypt, according to the National Committee for Clinical Laboratory Standards^27^.

Each uropathogen was freshly prepared and cultured on nutrient agar at 35–37 °C overnight. An inoculum of 1.5 × 10^8^ CFU/ml (equivalent to 0.5 McFarland) was prepared in sterile saline (0.85%) and swabbed over the surface of the prepared Mueller-Hinton agar plate. EEP and propolis fractions were prepared at a 100 mg/ml concentration. Wells were prepared in each plate using a sterile cork borer with a diameter of 8 mm and a volume of 100 µl of EEP, propolis fractions, honey (50% v/v), and DMSO (negative control) were dropped in each well. Also, cefotaxime (CTX 30), ciprofloxacin (CIP 5), and fluconazole 25 µg discs were used as positive controls, the zone of inhibition was measured, and results were interpreted according to the Clinical and Laboratory Standards Institute^28^. The plates were placed in the refrigerator for 1–2 h to allow the extracts to diffuse into the medium^9^. Then, the plates were incubated at 35–37 °C for 18–24 h, and the inhibition zone diameters (IZDs) were measured in mm. The experiment was performed in duplicate, and the mean ± standard error (SE) of the results was calculated.

#### Minimum inhibitory concentration (MIC) determination

The MIC value of EEP was evaluated using the broth microdilution method in the Regional Center for Mycology and Biotechnology, Cairo, Egypt, according to Helmy et al.^9^ study. At 96 well microtiter plates (BioTek, Winooski, VT, USA), an inoculum of each uropathogen suspension (1.5 × 10^8^ CFU/ml) was incubated with broth containing different concentrations of EEP (0.12–125 mg/ml) for 24 h at 37 °C. The MIC value was estimated by visual and spectroscopic methods by absorbance measurement at 620 nm. Control tubes without uropathogen (negative controls) and without EEP (positive controls) were used. The experiment was performed in duplicate, and the MIC ± standard error (SE) of the results was calculated.

### Antimicrobial synergism of the EEP-Honey mixture

Antimicrobial synergy testing of the EEP-Honey mixture against MDR uropathogens was performed using the agar well diffusion method and MIC determination, as previously described in the above sections. However, in determining the MIC, different concentrations of the EEP-Honey mixture were prepared (1:1 v/v) below the MIC value of EEP, and these mixtures were screened against MDR uropathogens as described above. The synergism was defined when the MIC value of the EEP-Honey mixture reduced the MIC value of EEP alone^29^, as all urinary pathogens were resistant to honey.

### Phytochemical analysis of propolis extract

**Fig. 1:**
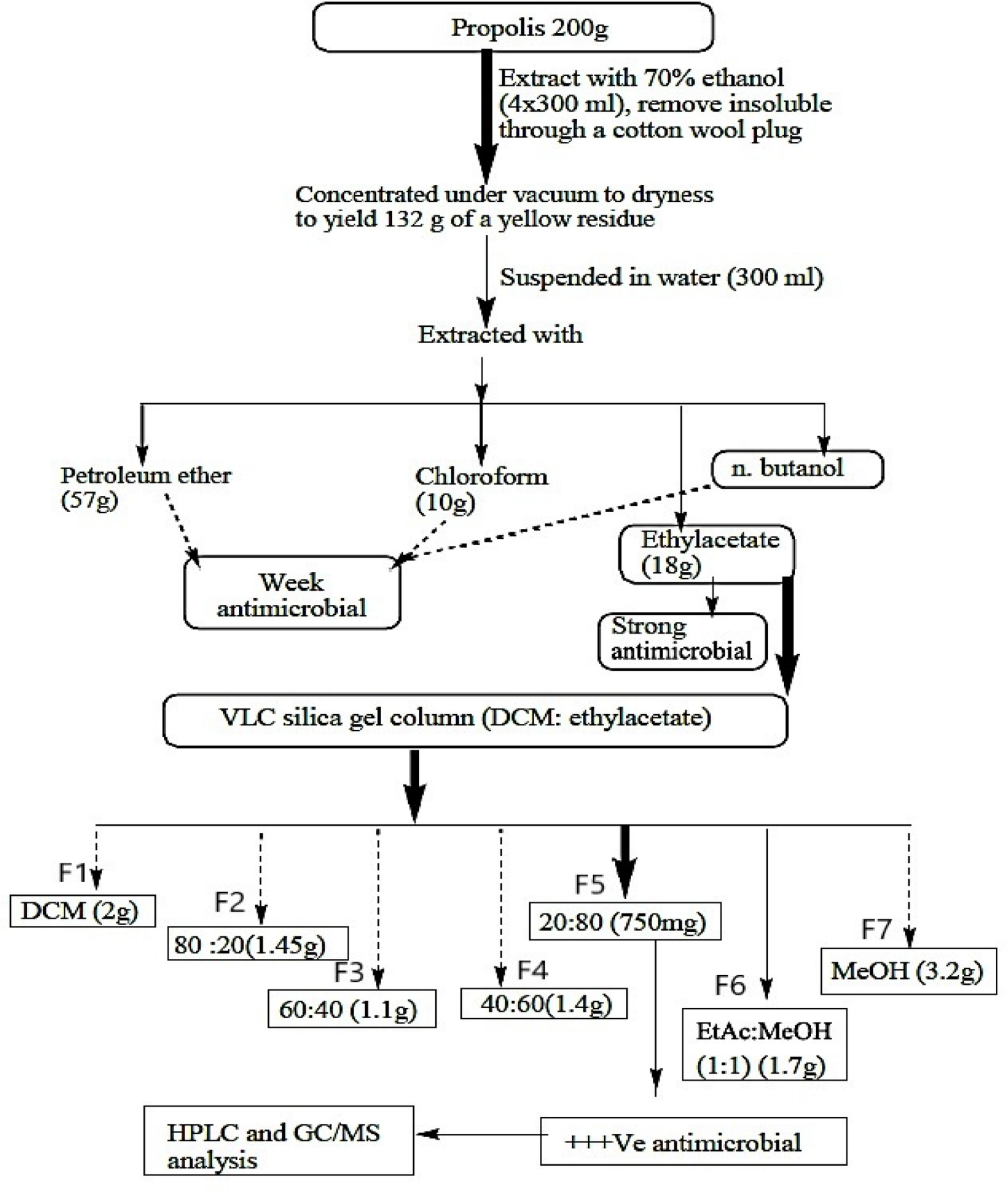
Propolis fractionation and purification of the most active antimicrobial substance. VLC: vacuum liquid chromatography, DCM: dichloromethane, EtAc: ethyl acetate, MeOH: methanol, GC-MS: gas chromatography-mass spectrometry, HPLC: high-performance liquid chromatography, +++ ve: strong positive activity, F1- F7: fraction 1- fraction 7.

Phytochemical analysis of propolis was performed at the Faculty of Pharmacy at Al-Azhar University for boys in Cairo, Egypt, according to Afsar et al.^30^, with some modifications, Fig. 1. About 200 g of propolis was extracted with 70% ethanol to obtain EEP. The dried EEP was re-suspended in 300 ml of water and subjected to successive liquid-liquid extraction using petroleum ether, chloroform, ethyl acetate, and n-butanol solvent systems, and their antimicrobial activities were studied. The ethyl acetate fraction (the highest in the antimicrobial activity) was subjected to further fractionation through vacuum liquid chromatography (VLC) on silica gel column and eluted with dichloromethane (DCM), dichloromethane (DCM): ethyl acetate (80:20, 60:40, 40:60, and 20:80 v/v), ethyl acetate (EtAc): methanol (MeOH) (1:1), and methanol (MeOH) to afford seven fractions in powder form, named (F1-F7) Fig. 1. The antimicrobial activity of the seven purified fractions was studied against MDR uropathogens, and the fraction with the highest antimicrobial activity was analyzed by gas-chromatography mass-spectrometry (GC-MS) and high-performance liquid chromatography (HPLC).

### Characterization of the most active propolis fraction

The most active fraction (F5) with the highest antimicrobial activity was analyzed by GC-MS and HPLC polyphenol analysis to predict the empirical chemical structure, molecular formula, and nomenclature of the most active compounds.

#### GC-MS analysis of propolis active fraction (F5)

The GC-MS system (Agilent Technologies) was equipped with a gas chromatograph (7890B) and mass spectrometer detector (5977 A) at Central Laboratories Network, National Research Centre; Cairo, Egypt. The active propolis fraction (F5) derivatizations were carried out using trimethylsilyl (TMS) derivatization and based on the optimized protocol described by Villas-Bôas et al.^31^. In summary, dried samples were re-suspended in 20 µL of pyridine and 100 µL of *N*-Methyl-*N*-(trimethylsilyl) trifluoroacetamide (MSTFA) and incubated in a dry block heater at 70 °C for 60 min. The GC was equipped with an HP-5MS column (30 m x 0.25 mm internal diameter and 0.25 *µ*m film thickness). Analyses were carried out using helium as the carrier gas at a flow rate of 1.0 ml/min at a splitless injection volume of 2 µl and the following temperature program: 50 °C for 10 min; rising at 8 °C/min to 300 °C and held for 10 min. The injector and detector were held at 280 °C and 220 °C, respectively. Mass spectra were obtained by electron ionization (EI) at 70 eV using a spectral range of 50–550 m/z and a solvent delay of 10 min. The mass temperature was 230 °C and Quad 150 °C. Identification of different constituents was determined by comparing the spectrum fragmentation pattern with those stored in Wiley and NIST Mass Spectral Library data.

#### HPLC analysis of propolis active fraction (F5)

HPLC polyphenol analysis of the most active fraction was carried out using an Agilent 1260 series. The separation was carried out using a Kromasil C18 column (4.6 mm x 250 mm i.d., 5 μm). The mobile phase consisted of water (A) and 0.05% trifluoroacetic acid in acetonitrile (B) at a flow rate of 1 ml/min. The mobile phase was programmed consecutively in a linear gradient as follows: 0 min (82% A); 0–5 min (80% A); 5–8 min (60% A); 8–12 min (60% A); 12–15 min (85% A) and 15–16 min (82% A). The injection volume was 10 µl for each of the sample solutions (15 mg/ml). The column temperature was maintained at 35 °C. Polyphenol compounds were assayed by external standard calibration at 280 nm. HPLC analysis was carried out in the Central Laboratories Network, National Research Centre, Cairo, Egypt^32^.

### Data analysis

Data were presented as mean, and standard error (SE) was determined. Data were normally distributed, and statistical analysis was performed using ANOVA one-way LSD tests. The software package CoStat for Windows version 6.45 was used for data analysis. A *P value* of < 0.05 was considered statistically significant for all analyses.

## Results

### Antimicrobial activity of EEP and its synergistic action with honey

In this study, the antimicrobial activity of EEP and its synergistic action with honey was determined by recording their IZDs and MIC values on MDR uropathogens. DMSO (negative control) was considered an inert solvent and didn’t report any inhibitory action on all MDR uropathogens. Also, cefotaxime (CTX 30), ciprofloxacin (CIP 5), and fluconazole 25-µg discs were used as positive controls and didn’t report inhibitory action on all MDR uropathogens since IZD less than 12 mm —for most antibiotics— was considered as resistant according to the Clinical and Laboratory Standards Institute breakpoints. Honey has not recorded any antimicrobial action, while EEP showed an inhibitory effect with variable action on all MDR uropathogens. Indeed, EEP showed higher activity against *C. albicans* and *S. saprophyticus* with IZDs of 21.67 and 21.33 mm and MIC values of 3.51 and 3.90 mg/ml, respectively, followed by *P. aeruginosa*,* K. pneumonia*, and *E.* coli with IZDs of 15.00, 13.83, and 12.58 mm, respectively, and MIC value ranged between 14.66 and 31.25 mg/ml; as shown in Table 1.

When propolis was mixed with honey, the mixture (EEP-Honey) showed a significant increase in its IZD in the range of 16.58–26.00 mm and a decrease in its MIC values compared to EEP alone against MDR uropathogens, as a good synergistic action was recorded. The results of the synergistic effect in Table 1 indicated that MDR Gram-negative uropathogens showed a decrease in their MIC value from 14.66 to 31.25 mg/ml in EEP to 1.95–7.81 mg/ml in the EEP-Honey mixture. Moreover, MDR *S. saprophyticus* and fluconazole-resistant *C. albicans* showed a decrease in the MIC values from 3.90 to 3.51 mg/ml in EEP to 0.98 and 0.95 mg/ml in the EEP-Honey mixture, respectively, when treated with (EEP-Honey) mixture.

The *p-values* ​​indicated that the EEP-Honey treatment option had a significantly greater antimicrobial effect than EEP alone across all tested pathogens. Thus, the statistical analysis suggests that honey enhances the antimicrobial properties of EEP, making EEP + Honey mixture a more effective treatment option.

**Table 1:** Antimicrobial activity of positive and negative controls, EEP, honey, and EEP-Honey mixture against MDR uropathogens.

pathogen	Mean IZD (mm) ± SE							MIC (mg/ml)	
	CTX 30	CIP 5	Flu 25	DMSO	EEP (100 mg/ml)	Honey (50%)	EEP + Honey	EEP	EEP + Honey
***E. coli***	0.00 ± 0.00	8.00 ± 0.58	NA	0.00 ± 0.00	12.85^c, B ^± 0.67	0.00 ± 0.00	16.58^c, A ^± 0.17	31.25^a, A ^± 1.52	7.81^c, B ^± 0.64
***K. pneumoniae***	0.00 ± 0.00	9.33 ± 0.67	NA	0.00 ± 0.00	13.83^bc, B ^± 0.44	0.00 ± 0.00	20.67^b, A ^± 0.58	15.63^b, A ^± 0.87	3.9^c, B ^± 0.51
***P. aeruginosa***	0.00 ± 0.00	0.00 ± 0.00	NA	0.00 ± 0.00	15.00^b, B ^± 0.58	0.00 ± 0.00	21.33^b, A ^± 0.33	14.66^b, A ^± 0.45	1.95^bc, B ^± 0.19
***S. saprophyticus***	7.00 ± 0.00	0.00 ± 0.00	NA	0.00 ± 0.00	21.33^a, B ^± 0.58	0.00 ± 0.00	25.67^a, A ^± 0.33	3.90^c, A ^± 0.37	0.98^b, B ^± 0.21
***C. albicans***	NA	NA	0.00 ± 0.00	0.00 ± 0.00	21.67^a, B ^± 0.33	0.00 ± 0.00	26.00^a, A ^± 0.67	3.51^c, A ^± 0.72	0.95^b, B ^± 0.48

SE = Standard error, CTX 30: Cefotaxime, CIP 5: Ciprofloxacin, Flu 25: Fluconazole, NA: Not applicable.

Different small letters indicate significant differences within the same column (*p-value* < 0.05).

Different capital letters indicate significant differences (*p-value* < 0.05) between columns in the same test.

A *p-value* < 0.05 was set as representing statistical significance for all analyses.

### Antimicrobial activity of the extracted substances from EEP using different solvent systems

**Fig. 2:**
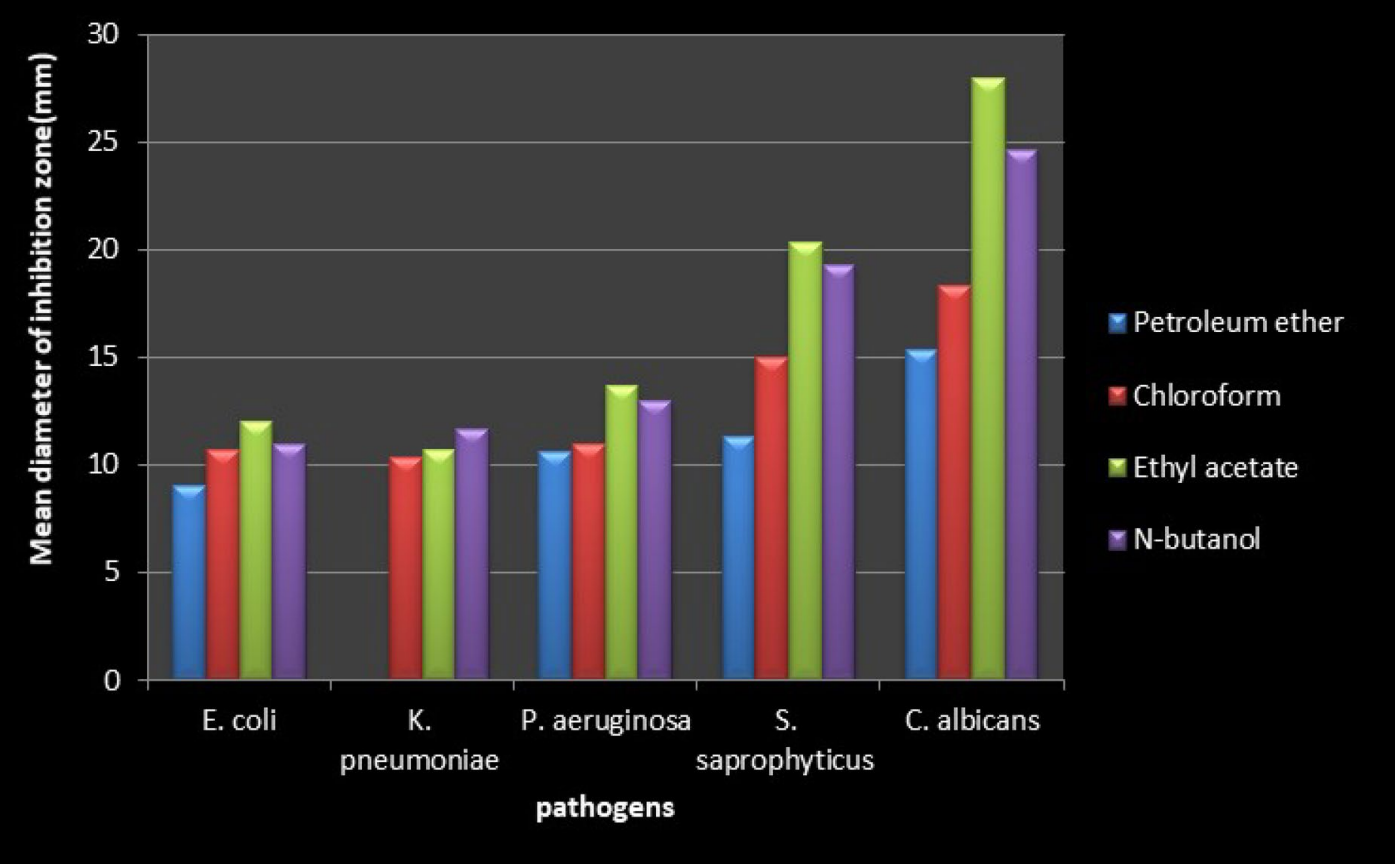
Effect of different solvent systems on antimicrobial activity of EEP against MDR uropathogens.

The data in Table 2; Fig. 2 indicated that, among all four solvent systems (petroleum ether, chloroform, ethyl acetate, and n-butanol), ethyl acetate was the best solvent for extracting the antimicrobial substance from EEP against MDR uropathogens (*p-value* < 0.05), except for *K. pneumonia*, followed by n-butanol, as it gave higher activity on this pathogen.

**Table 2:** Effect of different solvent systems on the antimicrobial activity of EEP against MDR uropathogens.

pathogen	Mean diameter of inhibition zone(mm) ± SE			
	Petroleum ether	Chloroform	Ethyl acetate	*N*-butanol
***E. coli***	9.00^c^ ± 0.67	10.67^b^ ± 0.67	**12.00**^**a**^ **± 0.00**	11.00^ab^ ± 0.00
***K. pneumoniae***	0.00^c^ ± 0.00	10.33^b^ ± 0.58	10.88^ab^ ± 0.33	**11.67**^**a**^ **± 0.67**
***P. aeruginosa***	10.58^b^ ± 0.17	11.00^b^ ± 0.17	**13.67**^**a**^ **± 0.67**	**13.00**^**a**^ **± 0.10**
***S. saprophyticus***	11.33^c^ ± 0.33	15.00^b^ ± 0.00	**20.33**^**a**^ **± 0.88**	**19.33**^**a**^ **± 0.58**
***C. albicans***	15.33^d^ ± 0.67	18.33^c^ ± 0.33	**28.00**^**a**^ **± 0.58**	24.67^b^ ± 0.33

Different small letters indicate significant differences (*p-value* < 0.05) between columns in the same test, representing the effect of the different solvent systems on the antimicrobial activity of EEP against each uropathogen. A *p-value* < 0.05 was set as representing statistical significance for all analyses.

### Antimicrobial activity of the fractions produced from VLC of Ethyl acetate EEP fraction

**Plate 1:**
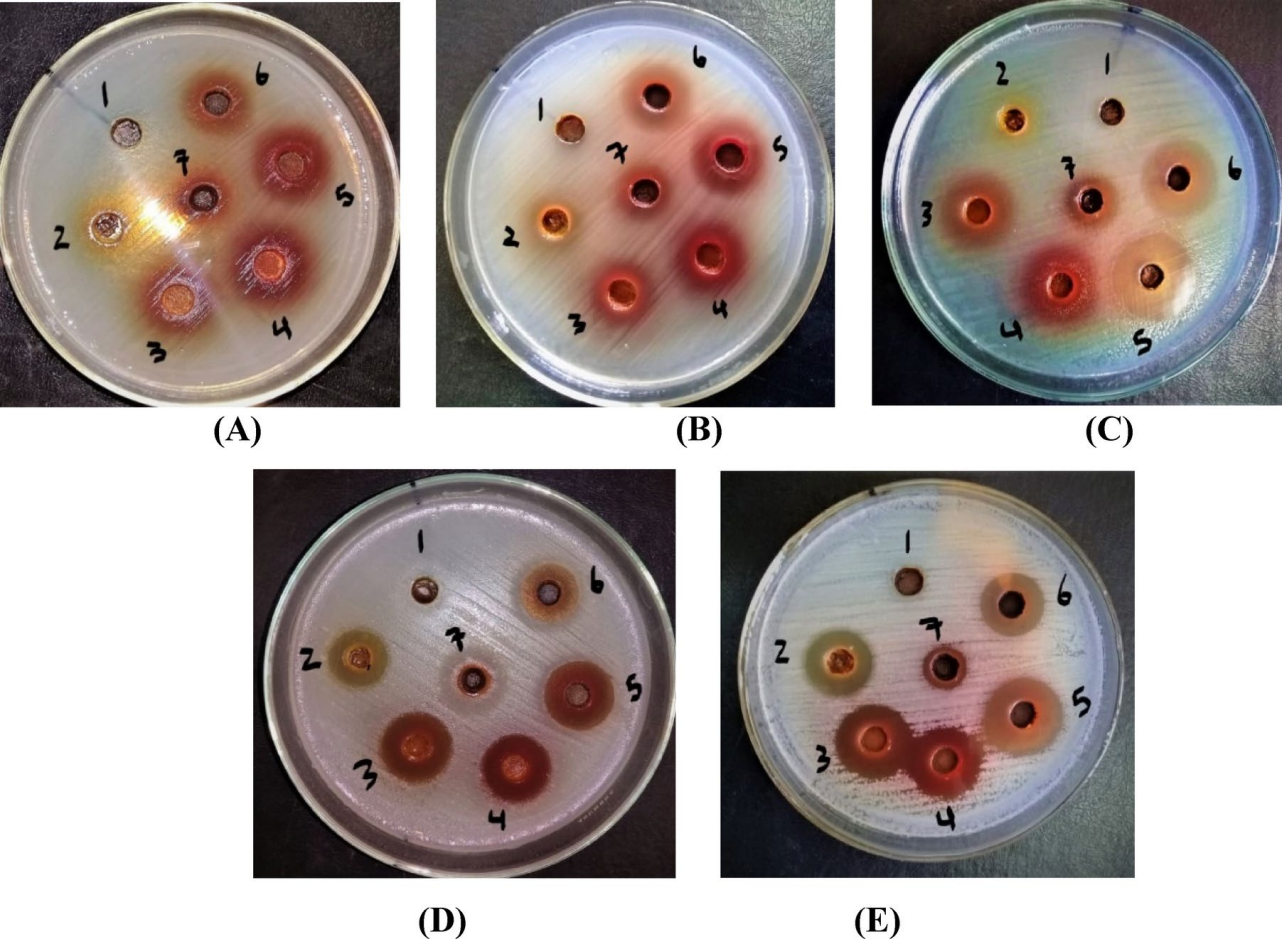
Plate’s photos reveal the antimicrobial activity of the fractions produced from VLC of ethyl acetate EEP fraction on MDR (**A**): *E. coli*, (**B**): *K. pneumonia*, (**C**): *P. aeruginosa*, (**D**): CON *S. saprophyticus*, and (**E**): *C. albicans.* Numbers from 1 to 7 indicated fraction numbers.

Using a VLC silica gel column, the ethyl acetate EEP fraction was separated into seven further fractions named F1, F2, F3, F4, F5, F6, and F7. Results in Table 3 and Plate 1 declared that the antimicrobial activity of the seven fractions was ordered as follows: F5 > F6 > F4 > F3 > F7 > F2 > F1. Fraction 5 (F5) that was eluted with dichloromethane (DCM): ethyl acetate (20:80) was the most active and gave significant antimicrobial activity among other fractions, with IZD of 30.33, 29.00, 27.67, 25.33, and 21.58 mm against the MDR *P. aeruginosa*, *E. coli*, *C. albicans*, *S. saprophyticus*, and *K. pneumoniae*, respectively. The p-values ​​indicated Fraction 5 (F5) had a significantly greater antimicrobial effect than other fractions across all tested pathogens.

**Table 3:** Antimicrobial activity of the fractions produced from VLC of ethyl acetate EEP fraction against MDR uropathogens.

Propolis Fractions	pathogens				
	E. coli	K. pneumoniae	*P*. aeruginosa	S. saprophyticus	C. albicans
	Mean diameter of inhibition zone(mm) ± SE				
**F1**	10.33^f^ ± 0.33	0.00^e^ ± 0.00	0.00^e^ ± 0.00	10.58^d^ ± 0.17	11.00^e^ ± 0.58
**F2**	12.00^e^ ± 0.58	12.00^d^ ± 0.00	13.33^d^ ± 0.33	20.33^b^ ± 0.67	20.00^c^ ± 0.00
**F3**	14.67^cd^ ± 0.33	13.33^cd^ ± 0.33	15.00^c^ ± 0.00	24.00^a^ ± 0.00	23.67^b^ ± 0.67
**F4**	15.33^c^ ± 0.67	14.00^c^ ± 0.33	15.33^c^ ± 0.33	22.00^b^ ± 0.58	23.00^b^ ± 0.00
**F5**	**29.00**^**a**^ **± 0.00**	**21.58**^**a**^ **± 1.17**	**30.33**^**a**^ **± 0.33**	**25.33**^**a**^ **± 0.67**	**27.67**^**a**^ **± 0.33**
**F6**	27.33^b^ ± 0.33	18.00^b^ ± 0.67	24.00^b^ ± 0.00	20.67^b^ ± 0.33	21.33^c^ ± 0.58
**F7**	14.00^d^ ± 0.00	13.00^cd^ ± 0.10	15.33^c^ ± 0.33	13.58^c^ ± 0.17	14.67^d^ ± 0.33

Different small letters indicate significant differences within the same column (*p-value* < 0.05). A *p-value* < 0.05 was set as representing statistical significance for all analyses.

### Characterization and identification of the most active Propolis fraction

Fraction 5 (F5) was the most active fraction and gave significant antimicrobial activity among other fractions and was consequently subjected to (GC-MS) and (HPLC) polyphenol analysis.

#### GC-MS analysis

**Fig. 3:**
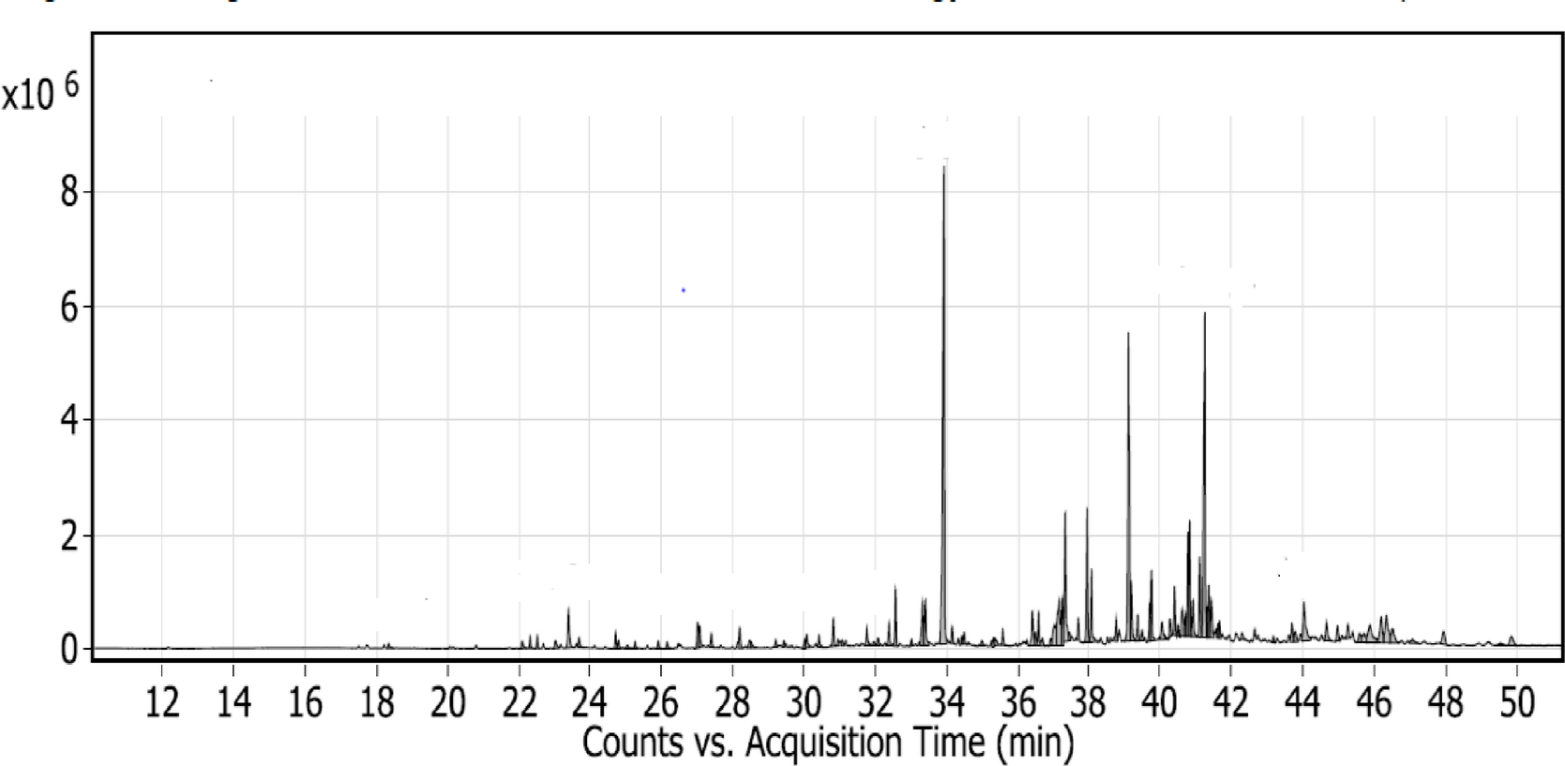
GC/MS analysis of the most active fraction of propolis.

The GC-MS analysis of the most active fraction allowed the detection of ninety compounds in different concentrations (99.1%), including phenolics, flavonoids, terpenoids, alkaloids, and organic acids, as shown in Table 4; Fig. 3. The major compounds present in the most active fraction of the propolis (F5) were identified as caffeic acid dimethyl ether (14.63%, compound 33, RT 33.925), monopalmitoylglycerol (9.79%, compound 54, RT 38.76), and 1,6-dihydroxy-8-methoxy-3-methylanthraquinone (9.12%, compound 69, RT 41.244), which contributed to the biological activity of propolis.

**Table 4:** Results of GC/MS analysis of the most active fraction of propolis.

Compound No.	Retention Time (RT)	compound	Formula	Area Sum %
1	18.338	Butoxyethanol	C_6_H_14_O_2_	0.16
2	22.092	Silanol	SiH_4_O	0.24
3	22.309	Cyclohexanediol	C_6_H_12_O_2_	0.27
4	22.515	Trans-cyclohexanediol	C_6_H_12_O_2_	0.24
5	23.025	Benzeneacetic acid	C_8_H_8_O_2_	0.28
6	23.391	Acetin	C_5_H_10_O_4_	1.37
7	24.713	Disiloxane, 1,3-bis(1,1 dimethylethyl)	C_12_H_30_OSi_2_	0.38
8	24.793	Resorcinol	C_6_H_6_O_2_	0.22
9	25.05	Butoxyacetate	C_10_H_20_O_3_	0.15
10	25.256	2*-Aminoisobutyric* acid	H_2_N-C(CH_3_)_2_-COOH	0.18
11	25.909	Oxyvaleric acid	CH_3_(CH_2_)_3_COOH	0.2
12	26.155	Kojic acid	C_6_H_6_O_4_	0.2
13	27.013	Cinnamic acid	C_9_H_8_O_2_	0.72
14	27.076	Pyrogallol	C_6_H_6_O_3_	0.59
15	27.396	Tyrosol	C_8_H_10_O_2_	0.32
16	28.192	-4-Hydroxybenzoic acid	C_7_H_6_O_3_	0.56
17	28.472	Dodecenoic acid	C_12_H_22_O_2_	0.28
18	29.204	Dimethoxybenzene	C_8_H_10_O_2_	0.18
19	29.433	10-Undecynoic acid	C_11_H_18_O_2_	0.15
20	30.017	Phloretic acid	C_9_H_10_O_3_	0.22
21	30.074	4-Methox benzoic acid	C_8_H_8_O_3_	0.41
22	30.417	Azelaic acid	C_9_H_16_O_4_	0.33
23	30.824	Protocatechoic acid	C_7_H_6_O_4_	0.7
24	30.95	p-methoxy Cinnamic acid	C_10_H_10_O_3_	0.19
25	31.07	Falcarinol	C_17_H_24_O	0.2
26	31.762	2,4-Dihydroxyacetophenone	C_8_H_8_O_3_	0.47
27	32.077	Hydroxydihydrosafrole	C_10_H_12_O_3_	0.25
28	32.386	Caffeic acid	C_9_H_8_O_4_	0.65
29	32.569	Gallic acid	C_7_H_6_O_5_	1.16
30	33.015	Ninhydrin	C_9_H_6_O_4_	0.16
31	33.324	Dimethyl caffeic acid	C_11_H_12_O_4_	1.4
32	33.404	Palmitic Acid	C_16_H_32_O_2_	1.29
33	33.925	Caffeic acid dimethyl ether	C_11_H_12_O_5_	**14.63**
34	34.16	beta.-Cholestane-alpha.,7.alpha.,12.alpha.,24.alpha.,25-pentol	C_27_H_44_O_5_	0.37
35	34.429	Benzothiophene-3-carbonitrile, 4,5,6,7-tetrahydro-2-(4-tert-butylbenzylidenamino	C_9_H_10_O_***2***_S	0.18
36	34.492	1,3-Bis(pentamethyldisilanyloxy)propane	C_17_H_20_O_6_S_2_	0.31
37	34.989	Beta- Eudesmol	C_15_H_26_O	0.17
38	35.316	13-Octadecenoic acid	C_18_H_34_O_2_	0.23
39	35.35	alpha.-D-Glucopyranoside	C_7_H_14_O_6_	0.29
40	35.573	Stearic acid	C_17_H_35_CO_2_H	0.37
41	36.408	Heneicosanoic acid	C_21_H_42_O_2_	1.06
42	36.494	5-dimethyl-6,8-dioxooctahydro-1 H-1,4-methanoinden-1-yl)propanoate	C_13_H_22_O_2_	0.42
43	36.586	− (3a,5-dimethyl-6,8-dioxooctahydro-1 H-1,4-methanoinden-1-yl)propanoate	C_16_H_30_O_2_	0.75
44	36.689	Vanillic acid	C_8_H_8_O_4_	0.29
45	36.918	1-ethyl-2-benzimidazolyl (2-methoxyphenyl)	C_11_H_14_O_3_	0.2
46	37.021	9-Octadecenamide	C_18_H_35_NO	1.14
47	37.158	Urocanic Acid	C_6_H_6_N_2_O_2_	2.42
48	37.244	Silane, diethyl(3-methylbutoxy)octadecyloxy	C_5_H_11_Cl_***3***_Si	1.31
49	37.33	t-Butyldimethyl(10-octylundec-10-enyloxy) silane	C_25_H_52_OSi	3.63
50	37.45	15-Isopropenyl-oxacyclopentadecan-2-one	C_20_H_38_O_2_Si	0.24
51	37.696	Hexakis(fluorodimethylsilyl)benzebe	C_18_H_36_F_6_Si_6_	0.5
52	37.942	1,4-benzenediacetonitrile, .alpha.,.alpha.‘-bis[[4-(diethylamino)-2-methoxyphenyl]methylene	C_34_H_38_N_4_O_2_	3.22
53	38.068	Linoleic *acid* (LA)	C_18_H_32_O_2_	1.65
54	38.76	Monopalmitoylglycerol	C_19_H_38_O_4_	**9.79**
55	39.184	3,7,11,15-tetramethylhexadecan-1,3-diol	C_20_H_*42*_O_2_	1.51
56	39.361	Erythro-Pentonic acid, 2-deoxy-3,4,5-tris-OH	C_17_H_42_O_5_	0.65
57	39.487	1-Monoferuloylglycerol	C_13_H_16_O_6_	0.26
58	39.75	5,8,11-Eicosatriynoic acid	C_20_H_28_O_2_	2.43
59	40.048	Tofisopam	C_22_H_26_N_2_O_4_	0.7
60	40.271	Arachidonic acid	C_20_H_24_O_2_	0.48
61	40.397	Silane, diethyloctyloxytetradecyloxy	C_26_H_56_O_2_Si	1.02
62	40.494	2-Monostearin	C_21_H_42_O_4_	0.23
63	40.614	1-Monooleoylglycerol	C_21_H_40_O_4_	1.07
64	40.706	Genistein	C_15_H_10_O_5_	0.55
65	40.774	Catechine	C_15_H_14_O_6_	2.33
66	40.826	Glycerol monostearate	C_21_H_42_O_4_	2.63
67	40.917	D-Glucopyranuronic acid	C_6_H_10_O_7_	0.99
68	41.106	[(2-{3,4-Bisoxy]phenyl}- oxy]-3,4-dihydro-2 H-chromen-7-yl-oxysilane	C_30_H_54_O_6_Si_5_	1.74
69	41.244	1,6-Dihydroxy-8-methoxy-3-methylanthraquinone	C_16_H_12_O_5_	**9.12**
70	41.318	(4-(1-(3,5-Dimethyl-4phenyl)-1,3-dimethylbutyl)-2,6-dimethylphenoxysilane	C_28_H_46_O_2_Si_2_	0.6
71	41.358	[(2-{3,4-Bisoxy]phenyl}-3,5-bis-3,4-dihydro-2 H-chromen-7-yl)oxy](trimethyl)silane	C_30_H_54_O_6_Si_***5***_	1.21
72	41.427	17.alpha.-Methyltestosterone	C_20_H_30_O_2_	1.14
73	41.621	4-Androsten-4-ol-3,17-dionel	C_19_H_26_O_3_	0.32
74	41.661	Monoketal adduct	C29H32O5	0.39
75	42.296	Saponarin	C_27_H_30_O_15_	0.24
76	43.172	3,5-Dihydroxybenzyl alcohol	C_7_H_8_O_3_	0.15
77	43.698	delta.(1)-Tetrahydrocannabinolic acid	C_22_H_30_O_4_	0.54
78	43.784	17-(1,5-Dimethylhexyl)-10,13-dimethyl-3-styrylhexadecahydrocyclopenta[a] phenanthren-2-one	C35H52O	0.43
79	44.036	-unknown		1.91
80	44.665	1,2,8-Trihydroxy-3-methoxy-6-methylanthraquinone	C_16_H_12_O_6_	0.66
81	44.974	Monoolein	C_21_H_40_O_4_	0.48
82	45.592	Homogentisic acid	C_8_H_8_O_4_	0.4
83	45.712	2,4-Imidazolidinedione, 5- oxy]phenyl]-3-methyl-5-phenyl	C_16_H_16_N_2_O_4_	0.54
84	45.884	Naringenin	C_15_H_12_O_5_	1.48
85	46.205	2-Oxabicyclo[3.3.0]octa-3,7-dien-6-one, 3-acetyl-4-methyl-1,5,7,8-tetrakis	C_22_H_42_O_7_	2.99
86	46.519	6-[Nonadecenyl]salicylic acid	C_26_H_42_O_3_	0.89
87	47.074	Octasiloxane, hexadecamethyl	C_16_H_50_O_7_	0.27
88	47.944	2-Monomyristin	C_17_H_34_O_4_	0.65
89	49.552	Heptasiloxane, tetradecamethyl ester	C_14_H_42_O_7_Si_7_	0.16
90	49.855	1-Monolinoleoylglycerol	C_27_H_54_O_4_	0.65
**Total**				**99.1**

#### HPLC analysis

**Fig. 4:**
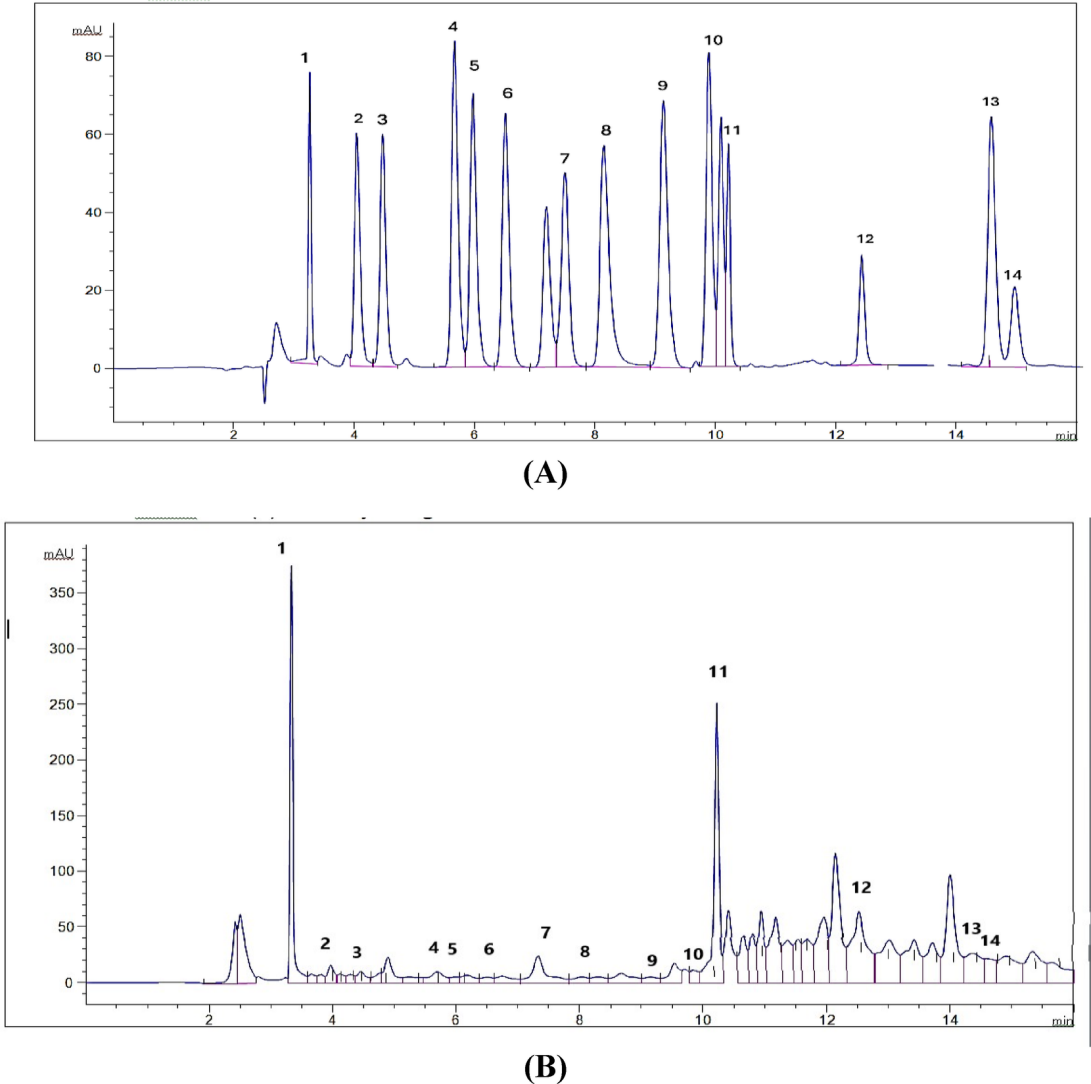
HPLC chromatogram of (**A**) reference standards polyphenol compounds and (**B**) fraction 5 in propolis with identified marker compounds. 1: gallic acid, 2: chlorogenic acid, 3: catechin, 4: methyl gallate, 5: coffeic acid, 6: syringic acid, 7: pyrocatechol, 8: ellagic acid, 9: coumaric acid, 10: vanillin, 11: naringenin, 12: taxifolin, 13: cinnamic acid, and 14: kaempferol.

HPLC polyphenol analysis of the most active fraction (F5) in propolis revealed the presence of 14 phenolic acids and flavonoid compounds in the range of 117.36–5657.66 µg/g. These compounds included 4 flavonoids (catechin, naringenin, taxifolin, and kaempferol) and 10 phenolic acids (gallic acid, chlorogenic acid, catechin, methyl gallate, caffeic acid, syringic acid, pyrocatechol, ellagic acid, coumaric acid, vanillin, and cinnamic acid). The biological activity of the propolis fraction contributed to the most abundant compounds, which included naringenin (5657.66 µg/g), gallic acid (5217.66 µg/g), taxifolin (5192.48 µg/g), pyrocatechol (2182.90 µg/g), and kaempferol (1029.27 µg/g), as shown in Table 5; Fig. 4.

**Table 5:** Results of HPLC analysis of fraction 5.

Peak No.	Retention time (RT)		Concentration (µg/g)	Identified compounds
	Standard	Test		
1.	3.263	3.334	**5217.66**	**Gallic acid**
2.	4.046	3.970	594.05	Chlorogenic acid
3.	4.475	4.460	1183.75	Catechin
4.	5.668	5.694	194.71	Methyl gallate
5.	5.974	5.993	121.32	Coffeic acid
6.	6.513	6.504	153.70	Syringic acid
7.	7.193	7.328	**2182.90**	**Pyrocatechol**
8.	8.147	8.301	336.62	Ellagic acid
9.	9.139	9.149	117.36	Coumaric acid
10.	9.894	9.848	154.83	Vanillin
11.	10.218	10.223	**5657.66**	**Naringenin**
12.	13.431	13.019	**5192.48**	**Taxifolin**
13.	14.332	14.590	322.58	Cinnamic acid
14.	14.715	14.907	**1029.27**	**Kaempferol**

## Discussion

Propolis (bee glue) is as old as honey and has been used by humans as a traditional medicine since antiquity. There are many records recommending its utilization in medication by the ancient Egyptians, Persians, and Romans^33^. The results of the in vitro antimicrobial assay indicated that EEP inhibited the growth of Gram-positive CON *S. saprophyticus* bacteria and *Candida albicans* yeast better than Gram-negative *P. aeruginosa*,* K. pneumonia*, and *E.* coli bacteria. This is explained by the presence of the outer membrane in the Gram-negative bacteria^14,34^. The low sensitivity of *E. coli* toward propolis has also been reported by many researchers, as this bacterium showed either very low sensitivity or a total lack of sensitivity to propolis^35,36^. Moreover, a previous study by Taher^37^ supported our results and reported that MDR *K. pneumoniae* was sensitive to Iraq EEP with an average inhibition zone of 12.6 mm at a concentration of 100 mg/ml, while another study by Al-Ani et al.^22^ found *P. aeruginosa* displayed high resistance to European propolis, which is in contrast to our results. In the current study, the MIC value of EEP on *C. albicans* and *S. saprophyticus* was 3.51 and 3.90 mg/ml, respectively, while against *P. aeruginosa*,* K. pneumonia*, and *E.* coli were in the range of 14.66–31.25 mg/ml, which were higher than a previous study of ours on Turkish propolis that reported MIC values in the range of 0.185-3.50 mg/ml on *K. pneumonia*, *P. aeruginosa*,* S. aureus*, and *C. albicans*^9^. On the other hand, previous studies have concluded that natural honey can inhibit the growth of some pathogenic bacteria with varying inhibition degrees^9,38,39^, while honey in our study showed no effect on the isolated pathogens, which may be due to the extreme resistance of urinary pathogens to antibiotics.

The addition of propolis to honey resulted in an increase in IZDs of EEP from 12.85 to 21.67 mm to 16.58–26.00 mm in the EEP-Honey mixture as well as a decrease in the MIC values of EEP from 3.51 to 31.25 mg/ml to 0.95–7.81 mg/ml in the EEP-Honey mixture, where significant synergy has been recorded. Basically, the antimicrobial synergism of propolis and honey may be related to the synergistic effect of their various flavonoids and phenolic compounds, which have been supported by previous studies^24,29,40^. In line with our findings, another study by Hamouda et al.^41^ reported the good antimicrobial effect of Egyptian fennel honey and EEP against 19 strains of methicillin-resistant *Staphylococcus aureus* (MRSA), where honey and EEP showed a synergistic effect when added together (MIC = 7.84 mg/ml).

Propolis fractionation was performed using a three-step sequential extraction: first with 70% ethanol, followed by the use of four solvent systems (petroleum ether, chloroform, ethyl acetate, and n-butanol), where ethyl acetate was the best solvent for the extraction of the antimicrobial substance from EEP. These results were supported by the study of Bouaroura et al.^42^ using Algerian propolis, as the ethyl acetate extract displayed increased contents of antimicrobial substances. Further, findings in the Chen et al.^43^ study reported the ethyl acetate fraction showed the highest total phenolic and flavonoid contents, which have potential antioxidant and antimicrobial activity, compared to the n-butanol fraction, chloroform fraction, and petroleum ether fractions. The standard protocols for chemical fractionation and bioactivity-guided chemical analysis were used to identify the bioactive ethyl acetate fraction, as it contained the highest polyphenol contents^44^. The third step was carried out using a VLC silica gel column, as the ethyl acetate EEP fraction was separated into seven further fractions named F1, F2, F3, F4, F5, F6, and F7, on which the pure F5 fraction gave the best antimicrobial activity against MDR uropathogens and was subsequently analyzed by GC-MS and HPLC.

The GC-MS analysis of the F5 fraction revealed the presence of 90 chemical compounds at different concentrations, including phenolic acids, flavonoids, terpenoids, alkaloids, steroids, organic acids, fatty acids, hydrocarbon esters, ketones, and sugars. These compounds act synergistically and are responsible for the therapeutical and pharmacological properties of propolis^12,45^. Polyphenol compounds (phenolic acids and flavonoids) are considered the main antimicrobial and bioactive antioxidant components in propolis^9,46^. In our study, caffeic acid esters are strong bactericidal agents and inhibit bacterial growth by disrupting membrane permeability or through oxidative stress mechanisms^47,48^. Also, 1,6-Dihydroxy-8-methoxy-3-methylanthraquinone and monopalmitoylglycerol were reported in propolis in Kurek-Górecka et al.^48^ and Shi et al.^49^. studies with anti-inflammatory properties. Further, 4-hydroxycinnamic acid is a derivative of cinnamic acid and disrupts the outer membrane of Gram-negative bacteria, causing cytoplasmic leakage^50^.

The HPLC analysis of the most active propolis fraction (F5) resulted in the presence of 14 polyphenol compounds, including naringenin, gallic acid, taxifolin, pyrocatechol, kaempferol, catechin, chlorogenic acid, methyl gallate, coffeic acid, syringic acid, ellagic acid, coumaric acid, vanillin, and cinnamic acid, which contributed to the antimicrobial activities of propolis. Previous studies on African, Asian, and European propolis concluded that propolis contains predominantly polyphenols such as naringenin, galangin, pinocembrin, apigenin, pinobanksin, quercetin, cinnamic acid and its esters, aromatic acids and their esters, kaempferol, chrysin, p-coumaric acid, cinnamyl caffeate, cinnamylidene acetic acid, and caffeic acid^46,51,52^. The composition of propolis varies depending on the plant’s origin, geographical location, and collection seasons^9,22^.

**Fig. 5:**
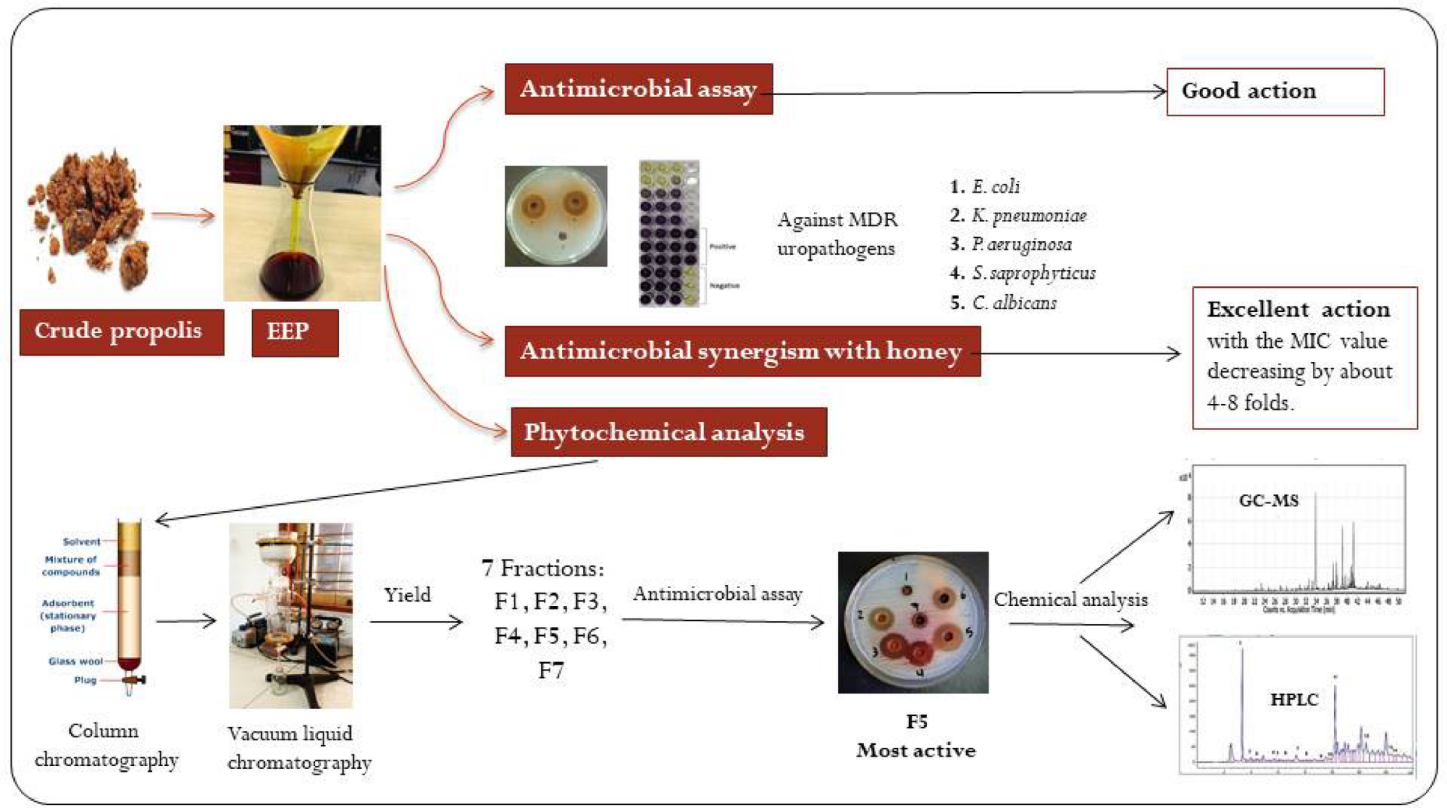
A Schematic drawing illustrating the design of the study and the important findings.

The high concentration of naringenin and taxifolin flavonoids in our study may be the main reason for the significant inhibitory effect of F5 fraction against MDR uropathogens, especially *P. aeruginosa* (IZD = 30.33 mm), where Vandeputte et al.^53^ and Shariati et al.^54^ studies reported the inhibitory effect of naringenin and taxifolin on the expression of various quorum sensing (QS) controlled genes in *P. aeruginosa* and thus reduce the virulence factors of pathogenic bacteria. Furthermore, naringenin possesses anti-staphylococcal activity^55,56^ and antibacterial properties against *E. coli*^56^. Likewise, some phenolic acids, such as cinnamic acid and its derivatives, have anti-QS activity and inhibit bacteria by damaging the cell membrane, inhibiting ATPases, cell division, and biofilm formation^57^. Also, other phenolic acids like ferulic, caffeic, and chlorogenic acids are effective in preventing bacterial adhesion^58,59^; while gallic acid inhibits efflux pump mechanisms in MDR *Staphylococcus* spp. and *E. coli* strains. The mixture of phenolic catechin and vanillic acids possesses antioxidant and antimicrobial synergy and is effective in preventing cell adhesion and biofilm formation in uropathogenic *E. coli* compared to single compounds or nitrofurantoin antibiotics^60^. Accordingly, the combination of many active components in propolis and their presence in various proportions prevents the occurrence of bacterial resistance^48,61,62^. Finally, the design of the study and the important findings were presented as a schematic drawing in Fig. 5.

## Conclusion

Over recent years, the increasing empiric treatment with antibiotics in UTIs and the emergence of MDR urinary pathogens have stimulated the use of traditional medicine and attempts to improve it for therapeutic use, such as propolis, honey, and their mixtures. In the current study, propolis inhibited all MDR uropathogens, while honey did not. Interestingly, the combination of propolis and honey showed a great synergistic effect with a significant increase in the antimicrobial activity of propolis, which was expressed by reducing the MIC value of EEP for all uropathogens by approximately 4–8 folds. Propolis fractionation was carried out, as ethyl acetate was the best solvent to extract the antimicrobial substances from EEP. The ethyl acetate EEP fraction was further fractionated using a VLC silica gel column to get seven fractions, of which the F5 fraction was the most active with the best antimicrobial activity against MDR uropathogens and was analyzed by GC-MS and HPLC. HPLC analysis of the polyphenols in the F5 fraction of propolis revealed the presence of 14 phenolic acids and flavonoid compounds, where the most abundant compounds were naringenin, gallic acid, taxifolin, and pyrocatechol. GC-MS analysis indicated the presence of many constituents in different concentrations, including phenolic acids, flavonoids, terpenoids, and alkaloids, which are responsible for the biological and pharmaceutical properties of propolis. Thus, the combination of many active ingredients in propolis and their presence in various proportions prevents bacterial resistance.

## Data Availability

All information created or analyzed during the present study are included in the manuscript.

## Abbreviations

CON: Coagulase negative
EEP: Ethanolic extract of propolis
ESBL: Extended spectrum β-lactam antibiotic
F1: F7:Fraction 1:fraction 7
GC-MS: Gas chromatography-mass spectrometry
HPLC: High-performance liquid chromatography
IZD: Inhibition zone diameter
MDR: Multidrug-resistant
MIC: Minimum inhibitory concentration
MRSA: Methicillin-resistant Staphylococcus aureus
RT: Retention time
SE: Standard error
UTI: Urinary tract infection
VLC: Vacuum liquid chromatography

## References

[1] McLellan, L. K. & Hunstad, D. A. Urinary tract infection: pathogenesis and outlook. *Trends Mol. Med.* **22** (11), 946–957. 10.1016/j.molmed.2016.09.003 (2016). 27692880 10.1016/j.molmed.2016.09.003 PMC5159206

[2] Mlugu, E. M., Mohamedi, J. A., Sangeda, R. Z. & Mwambete, K. D. Prevalence of urinary tract infection and antimicrobial resistance patterns of uropathogens with biofilm forming capacity among outpatients in Morogoro, Tanzania: a cross-sectional study. *BMC Infect. Dis.* **23** (1), 660. 10.1186/s12879-023-08641-x (2023). 37798713 10.1186/s12879-023-08641-x PMC10557311

[3] Galiczewski, J. M. & Shurpin, K. M. An intervention to improve the catheter associated urinary tract infection rate in a medical intensive care unit: direct observation of catheter insertion procedure. *Intensive Crit. Care Nurs.* **40**, 26–34. 10.1016/j.iccn.2016.12.003 (2017). 28237090 10.1016/j.iccn.2016.12.003

[4] Waller, T. A., Pantin, S. A., Yenior, A. L. & Pujalte, G. G. Urinary tract infection antibiotic resistance in the united States. *Prim. Care: Clin. Office Pract.* **45** (3), 455–466. 10.1016/j.pop.2018.05.005 (2018). 10.1016/j.pop.2018.05.005 30115334

[5] Li, W. et al. Rapid identification and antimicrobial susceptibility testing for urinary tract pathogens by direct analysis of urine samples using a MALDI-TOF MS-based combined protocol. *Front. Microbiol.* **10**, 458039. 10.3389/fmicb.2019.01182 (2019). 10.3389/fmicb.2019.01182 PMC6560049 31231323

[6] Helmy, A. K., Sidkey, N. M., El-Badawy, R. E. & Hegazi, A. G. Emergence of microbial infections in some hospitals of Cairo, Egypt: studying their corresponding antimicrobial resistance profiles. *BMC Infect. Dis.* **23** (1), 424. 10.1186/s12879-023-08397-4 (2023). 37349674 10.1186/s12879-023-08397-4 PMC10286371

[7] World Health Organization (WHO). *Antibacterial Agents in Clinical Development: an Analysis of the Antibacterial Clinical Development Pipeline, Including Tuberculosis* (World Health Organization, 2017). https://iris.who.int/handle/10665/258965WHO/EMP/IAU/2017.12.

[8] D’Andrea, M. M., Fraziano, M., Thaller, M. C. & Rossolini, G. M. The urgent need for novel antimicrobial agents and strategies to fight antibiotic resistance. *Antibiotics* **8** (4), 254. 10.3390/antibiotics8040254 (2019). 31817707 10.3390/antibiotics8040254 PMC6963704

[9] Helmy, A. K., Hegazi, A. G. & Sidkey, N. M. Antimicrobial activity of some honeybee products on Multidrug-Resistant secondary microbial infection from COVID-19 patients. *Egypt J. Hosp. Med.* **92** (1). 10.21608/EJHM.2023.313899 (2023).

[10] Bankova, V. Chemical diversity of propolis and the problem of standardization. *J. Ethnopharmacol.* **100** (1–2), 114–117. 10.1016/j.jep.2005.05.004 (2005). 15993016 10.1016/j.jep.2005.05.004

[11] Tosic, S., Stojanovic, G., Mitic, S., Pavlovic, A. & Alagic, S. Mineral composition of selected Serbian propolis samples. *J. Apic. Sci.* **61** (1), 5–15. 10.1515/jas-2017-0001 (2017).

[12] Anjum, S. I. et al. Composition and functional properties of propolis (bee glue): A review. *Saudi J. Biol. Sci.* **26** (7), 1695–1703. 10.1016/j.sjbs.2018.08.013 (2019). 31762646 10.1016/j.sjbs.2018.08.013 PMC6864204

[13] Irigoiti, Y. et al. The use of propolis as a functional food ingredient: A review. *Trends Food Sci. Technol.* **115**, 297–306. 10.1016/j.tifs.2021.06.041 (2021).

[14] Sa-Eed, A. et al. In vitro antimicrobial activity of crude propolis extracts and fractions. *FEMS Microbes*. **4**10.1093/femsmc/xtad010 (2023). 10.1093/femsmc/xtad010 PMC10165684 37333437

[15] Yosri, N. et al. Anti-viral and Immunomodulatory properties of propolis: chemical diversity, Pharmacological properties, preclinical and clinical applications, and in Silico potential against SARS-CoV-2. *Foods* **10** (8), 1776. 10.3390/foods10081776 (2021). 34441553 10.3390/foods10081776 PMC8391193

[16] Negri, G. et al. Antiviral activity of red propolis against herpes simplex virus-1. *Brazilian J. Pharm. Sci.* **60**, e23746. 10.1590/s2175-97902024e23746 (2024).

[17] Abduh, M. Y., Shafitri, T. R. & Elfahmi, E. Chemical profiling, bioactive compounds, antioxidant, and anti-inflammatory activities of Indonesian propolis extract produced by Tetragonula laeviceps. *Heliyon* **10** (19). 10.1016/j.heliyon.2024.e38736 (2024). 10.1016/j.heliyon.2024.e38736 PMC11471232 39397935

[18] Nichitoi, M. M. et al. Polyphenolics profile effects upon the antioxidant and antimicrobial activity of propolis extracts. *Sci. Rep.* **11** (1), 20113. 10.1038/s41598-021-97130-9 (2021). 34635677 10.1038/s41598-021-97130-9 PMC8505647

[19] Shaker, S. A. et al. Propolis-loaded nanostructured lipid carriers halt breast cancer progression through miRNA-223 related pathways: an in-vitro/in-vivo experiment. *Sci. Rep.* **13** (1), 15752. 10.1038/s41598-023-42709-7 (2023). 37735586 10.1038/s41598-023-42709-7 PMC10514043

[20] Saddiq, A. A. & Danial, E. N. Effect of Propolis as a food additive on the growth rate of the beneficial bacteria. *Main Group Chem.* **13** (3), 223–332. 10.3233/MGC-140135 (2014).

[21] Santos-Buelga, C. & González-Paramás, A. M. Bee products-chemical and biological properties. *Chemical Composition of Honey. Springer* 43–82 https://link.springer.com/book/10.1007/978-3-319-59689-1 (2017).

[22] Al-Ani, I., Zimmermann, S., Reichling, J. & Wink, M. Antimicrobial activities of European propolis collected from various geographic origins alone and in combination with antibiotics. *Medicines* **5** (1), 2. 10.3390/medicines5010002 (2018). 29301368 10.3390/medicines5010002 PMC5874567

[23] El Menyiy, N., Bakour, M., El Ghouizi, A., El Guendouz, S. & Lyoussi, B. Influence of geographic origin and plant source on physicochemical properties, mineral content, and antioxidant and antibacterial activities of Moroccan Propolis. *Int. J. Food Sci.* **2021** (1), 5570224. 10.1155/2021/5570224 (2021). 33791359 10.1155/2021/5570224 PMC7997750

[24] Salatino, A. Perspectives for uses of propolis in therapy against infectious diseases. *Molecules* **27** (14), 4594. 10.3390/molecules27144594 (2022). 35889466 10.3390/molecules27144594 PMC9320184

[25] Pincus, D. H. Microbial identification using the bioMérieux Vitek^®^ 2 system. Encyclopedia of Rapid Microbiological Methods. Bethesda, MD: *Parenteral Drug Association.* 1–32 https://store.pda.org/tableofcontents/ermm_v2_ch01.pdf (2006).

[26] Barry, A. et al. Quality control limits for fluconazole disk susceptibility tests on Mueller-Hinton agar with glucose and methylene blue. *J. Clin. Microbiol.* **41** (7), 3410–3412. 10.1128/jcm.41.7.3410-3412.2003 (2003). 12843106 10.1128/JCM.41.7.3410-3412.2003 PMC165306

[27] National Committee for Clinical Laboratory Standards (NCCLS). *Methods for Dilution Antimicrobial Susceptibility Tests of bacteria that Grow Aerobically: Approved Standard M100-S12* (NCCLS, 2002).

[28] Clinical and Laboratory Standards Institute. Performance standards for antimicrobial susceptibility testing. 30th ed. CLSI supplement M100. Wayne, PA: CLSI. Available from: https://www.nih.org.pk/wp-content/uploads/2021/02/CLSI-2020.pdf (2020).

[29] Noori, A. L., Al-Ghamdi, A., Ansari, M. J., Al-Attal, Y. & Salom, K. Synergistic effects of honey and propolis toward drug multi-resistant Staphylococcus aureus, Escherichia coli and Candida albicans isolates in single and polymicrobial cultures. *Int. J. Med. Sci.* **9** (9), 793. 10.7150/ijms.4722 (2012). 23136543 10.7150/ijms.4722 PMC3491439

[30] Afsar, T. et al. Bioassay-guided isolation and characterization of lead antimicrobial compounds from Acacia Hydaspica plant extract. *AMB Express*. **12** (1), 156. 10.1186/s13568-022-01501-y (2022). 36520322 10.1186/s13568-022-01501-y PMC9755427

[31] Villas-Bôas, S. G., Noel, S., Lane, G. A., Attwood, G. & Cookson, A. Extracellular metabolomics: a metabolic footprinting approach to assess fiber degradation in complex media. *Anal. Biochem.* **349** (2), 297–305. 10.1016/j.ab.2005.11.019 (2006). 16356465 10.1016/j.ab.2005.11.019

[32] Abdel-Aziz, A. W., Elwan, N. M., Shaaban, R. S., Osman, N. S. & Mohamed, M. A. HighPerformance liquid chromatographyfingerprint analyses, in vitro cytotoxicity, antimicrobial and antioxidant activities of the extracts of Ceiba speciosa growing in Egypt. *Egypt. J. Chem.* **64** (4), 1831–1843. 10.21608/ejchem.2021.58716.3267 (2021).

[33] Kuropatnicki, A. K., Szliszka, E. & Krol, W. Historical aspects of propolis research in modern times. *Evidence-Based Complement. Altern. Med.* **2013** (1), 964149. 10.1155/2013/964149 (2013). 10.1155/2013/964149 PMC3655583 23710243

[34] Alegun, O., Pandeya, A., Cui, J., Ojo, I. & Wei, Y. Donnan potential across the outer membrane of gram-negative bacteria and its effect on the permeability of antibiotics. *Antibiotics* **10** (6), 701. 10.3390/antibiotics10060701 (2021). 34208097 10.3390/antibiotics10060701 PMC8230823

[35] Kujumgiev, A. et al. Antibacterial, antifungal and antiviral activity of propolis of different geographic origin. *J. Ethnopharmacol.* **64** (3), 235–240. 10.1016/s0378-8741(98)00131-7 (1999). 10363838 10.1016/s0378-8741(98)00131-7

[36] Gonzalez, B. E. et al. Severe Staphylococcal sepsis in adolescents in the era of community-acquired methicillin-resistant Staphylococcus aureus. *Pediatrics* **115** (3), 642–648. 10.1542/peds.2004-2300 (2005). 15741366 10.1542/peds.2004-2300

[37] Taher, N. M. Synergistic effect of Propolis extract and antibiotics on Multi-Resist Klebsiella Pneumoniae strain isolated Fom wound. *Adv. Life Sci. Technol.* **43**. https://core.ac.uk/reader/234687340 (2016).

[38] Johnston, M., McBride, M., Dahiya, D., Owusu-Apenten, R. & Nigam, P. S. Antibacterial activity of Manuka honey and its components: an overview. *AIMS Microbiol.* **4** (4), 655. 10.3934/microbiol.2018.4.655 (2018). 31294240 10.3934/microbiol.2018.4.655 PMC6613335

[39] Prudence, I. A., Celestin, M. P., Hiberte, M., Josue, I. & Eric, S. The effect of honey on Bacteria isolated from urinary tract infections among patients attending ruhengeri referral hospital. *J. Drug Delivery Ther.* **14** (4), 10–13. 10.22270/jddt.v14i4.6104 (2024).

[40] Vică, M. L. et al. Antibacterial activity of propolis extracts from the central region of Romania against neisseria gonorrhoeae. *Antibiotics* **10** (6), 689. 10.3390/antibiotics10060689 (2021). 34201299 10.3390/antibiotics10060689 PMC8226552

[41] Hamouda, S. M., Abd El Rahman, M. F., Abdul-Hafeez, M. M. & Gerges, A. E. Egyptian fennel honey and/or propolis against MRSA harboring both MecA & IcaA genes. *Int. J. Complement. Altern. Med.* **11**, 180–185. 10.15406/ijcam.2018.11.00392 (2018).

[42] Bouaroura, A. et al. Phytochemical investigation of phenolic constituents and in vitro evaluation of antioxidant activity of five Algerian Propolis. *Curr. Bioact. Compd.* **17** (8), 79–87. 10.2174/1573407216999201231200041 (2021).

[43] Chen, X., He, X., Sun, J. & Wang, Z. Phytochemical composition, antioxidant activity, α-glucosidase and acetylcholinesterase inhibitory activity of Quinoa extract and its fractions. *Molecules* **27** (8), 2420. 10.3390/molecules27082420 (2022). 35458616 10.3390/molecules27082420 PMC9032577

[44] Elnakady, Y. A. et al. Characteristics, chemical compositions and biological activities of propolis from Al-Bahah, Saudi Arabia. *Sci. Rep.* **7** (1), 41453. 10.1038/srep41453 (2017). 28165013 10.1038/srep41453 PMC5292687

[45] Mouhoubi-Tafinine, Z., Ouchemoukh, S. & Tamendjari, A. Antioxydant activity of some Algerian honey and propolis. *Ind. Crops Prod.* **88**, 85–90. 10.1016/j.indcrop.2016.02.033 (2016).

[46] Nada, A. A. et al. Synergistic effect of potential alpha-amylase inhibitors from Egyptian propolis with acarbose using in Silico and in vitro combination analysis. *BMC Complement. Med. Ther.* **24** (1), 65. 10.1186/s12906-024-04348-x (2024). 38291462 10.1186/s12906-024-04348-x PMC10826043

[47] Collins, W., Lowen, N. & Blake, D. J. Caffeic acid esters are effective bactericidal compounds against Paenibacillus larvae by altering intracellular oxidant and antioxidant levels. *Biomolecules* **9** (8), 312. 10.3390/biom9080312 (2019). 31357646 10.3390/biom9080312 PMC6722690

[48] Kurek-Górecka, A. et al. Comparison of the antioxidant activity of propolis samples from different geographical regions. *Plants* **11** (9), 1203. 10.3390/plants11091203 (2022). 35567206 10.3390/plants11091203 PMC9104821

[49] Shi, H. et al. Isolation and characterization of five glycerol esters from Wuhan propolis and their potential anti-inflammatory properties. *J. Agric. Food Chem.* **60** (40), 10041–10047. 10.1021/jf302601m (2012). 22978445 10.1021/jf302601m

[50] Guzman, J. D. Natural cinnamic acids, synthetic derivatives and hybrids with antimicrobial activity. *Molecules* **19** (12), 19292–19349. 10.3390/molecules191219292 (2014). 25429559 10.3390/molecules191219292 PMC6271800

[51] Huang, S., Zhang, C. P., Wang, K., Li, G. Q. & Hu, F. L. Recent advances in the chemical composition of propolis. *Molecules* **19** (12), 19610–19632. 10.3390/molecules191219610 (2014). 25432012 10.3390/molecules191219610 PMC6271758

[52] Machado, C. S., Finger, D., Caetano, I. K. & Torres, Y. R. Multivariate GC-MS data analysis of the apolar fraction of brown propolis produced in Southern Brazil. *J. Apic. Res.* **14**, 1–2. 10.1080/00218839.2023.2212487 (2023).

[53] Vandeputte, O. M. et al. The Flavanone naringenin reduces the production of quorum sensing-controlled virulence factors in Pseudomonas aeruginosa PAO1. *Microbiology* **157** (7), 2120–2132. 10.1099/mic.0.049338-0 (2011). 21546585 10.1099/mic.0.049338-0

[54] Shariati, A. et al. Inhibitory effect of natural compounds on quorum sensing system in Pseudomonas aeruginosa: a helpful promise for managing biofilm community. *Front. Pharmacology.* **15**, 1350391 10.3389/fphar.2024.1350391 (2024). 10.3389/fphar.2024.1350391 PMC11019022 38628638

[55] do Nascimento, T. G. et al. Comprehensive multivariate correlations between climatic effect, metabolite-profile, antioxidant capacity and antibacterial activity of Brazilian red propolis metabolites during seasonal study. *Sci. Rep.* **9** (1), 18293 10.1038/s41598-019-54591-3 (2019). 10.1038/s41598-019-54591-3 PMC6893030 31797960

[56] Dantas, D. M. et al. Naringenin as potentiator of Norfloxacin efficacy through Inhibition of the NorA efflux pump in Staphylococcus aureus. *Microb. Pathog.* **107504**10.1016/j.micpath.2025.107504 (2025). 10.1016/j.micpath.2025.107504 40154849

[57] Vasconcelos, N. G., Croda, J. & Simionatto, S. Antibacterial mechanisms of cinnamon and its constituents: A review. *Microb. Pathog.* **120**, 198–203. 10.1016/j.micpath.2018.04.036 (2018). 29702210 10.1016/j.micpath.2018.04.036

[58] Borges, A., Saavedra, M. J. & Simões, M. The activity of ferulic and Gallic acids in biofilm prevention and control of pathogenic bacteria. *Biofouling* **28** (7), 755–767. 10.1080/08927014.2012.706751 (2012). 22823343 10.1080/08927014.2012.706751

[59] Gupta, P., Song, B., Neto, C. & Camesano, T. A. Atomic force microscopy-guided fractionation reveals the influence of cranberry phytochemicals on adhesion of Escherichia coli. *Food Funct.* **7** (6), 2655–2666. 10.1039/C6FO00109B (2016). 27220364 10.1039/c6fo00109b

[60] Bernal-Mercado, A. T. et al. Comparison of single and combined use of Catechin, Protocatechuic, and vanillic acids as antioxidant and antibacterial agents against uropathogenic Escherichia coli at planktonic and biofilm levels. *Molecules* **23** (11), 2813. 10.3390/molecules23112813 (2018). 30380712 10.3390/molecules23112813 PMC6278301

[61] Pamplona-Zomenhan, L. C., Pamplona, B. C., Silva, C. B., Marcucci, M. C. & Mimica, L. M. Evaluation of the in vitro antimicrobial activity of an ethanol extract of Brazilian classified propolis on strains of Staphylococcus aureus. *Brazilian J. Microbiol.* **42**, 1259–1264. 10.1590/S1517-83822011000400002 (2011). 10.1590/S1517-83822011000400002 PMC3768705 24031749

[62] De Rossi, L., Rocchetti, G., Lucini, L., & Rebecchi, A. Antimicrobial potential of polyphenols: mechanisms of action and microbial responses-a narrative review. *Antioxidants* **14** (2), 200 10.3390/antiox14020200 (2025). 10.3390/antiox14020200 PMC11851925 40002386

